# Xingbi Gel Ameliorates Allergic Rhinitis by Regulating IFN-γ Gene Promoter Methylation in CD4+ T Cells *via* the ERK-DNMT Pathway

**DOI:** 10.3389/fsurg.2020.619053

**Published:** 2021-02-15

**Authors:** Si Ai, Yueyong Lin, Jian Zheng, Xiangli Zhuang

**Affiliations:** ^1^The Affiliated People's Hospital of Fujian University of Traditional Chinese Medicine, Fuzhou, China; ^2^No. 900 Hospital of the Joint Logistics Support Force of the Chinese People's Liberation Army, Fuzhou, China; ^3^Fujian University of Traditional Chinese Medicine, Fuzhou, China

**Keywords:** allergic rhinitis, Xingbi gel, methylation, ERK signaling pathway, CD4+ T cells

## Abstract

Allergic rhinitis (AR) is a common, non-infectious, chronic nasal mucosal disease primarily mediated by immunoglobulin E (IgE) following allergen exposure. Currently, studies on AR mainly focus on cytokines, IgE and its receptors, basophils, eosinophils, mast cells, and related genes. Among these, an imbalance between T helper (Th) 1 and Th2 cells is considered an important mechanism underlying AR pathogenesis. The most important cytokines in AR are interleukin (Il)-4 and interferon gamma (IFN-γ) which are secreted by Th2 and Th1 cells, respectively. Il-4 and IFN-γ are antagonistic to each other in regulating IgE synthesis. In this study, the expression of extracellular signal-regulated protein kinase (ERK) 1/2 and its phosphorylation from p-ERK1/2, were significantly increased in a cluster of differentiation of 4+ T cells of AR mice, suggesting that the ERK signaling pathway in these cells is involved in the occurrence and development of AR. This result also implies an enhanced expression of deoxyribonucleic acid methyltransferases (DNMTs). To verify the relationship between ERK signaling and DNMT expression, AR mice were treated with PD98059, a specific inhibitor of the ERK1/2 signaling pathway. The results revealed that perturbations in ERK signaling were significantly positively correlated with the downregulation of DNMT1 expression. Pharmacological intervention is key to treating AR. This study demonstrated that Xingbi gel intervention affected both serum IgE levels and AR behavior scores in mice. Based on its effects on IFN-γ gene expression, the regulation of Th1/Th2 balance, and the ERK signaling pathway, research on the effects of Xingbi gel on AR may provide new avenues in its prevention and treatment.

## Introduction

In recent years, the incidence of allergic rhinitis (AR) has been increasing annually, with serious effects on the quality of life. AR is a non-infectious inflammatory disease of the nasal mucosa that, following exposure to specific allergens, is caused by the release of the inflammatory mediator immunoglobulin E (IgE) and the activation of various immune cells. Its pathology is primarily characterized by eosinophil and mast cell aggregation in the nasal mucosa. The clinical manifestations are mainly nasal congestion, runny nose, sneezing, and nasal itching (1, 2). Epidemiological studies have reported that the incidence of AR is gradually increasing in developed countries and currently affects up to 40% of people worldwide (3). Interleukin (IL)-4, also known as B cell growth factor, is instrumental in AR pathogenesis and is the main factor in T helper (Th)2 cell differentiation. Interferon gamma (IFN-γ), the defining factor of Th1 cells, can inhibit Th2 cell proliferation and activation, reduce IL-4 production and antagonize its physiological effects, and prevent IgE synthesis (4). Indeed, Il-4 and IFN-γ have antagonistic effects on IgE synthesis (5).

Wang et al. (6) compared peripheral blood mononuclear cells between patients with AR and those with nasal polyps. In AR patients, IL-4 expression levels were increased, while INF-γ secretion was minimal, suggesting that Th0 cells were differentiating into Th2 cells. In patients with nasal polyps, the Th1/Th2 balance was disrupted; levels of both were increased. IFN-γ attenuates IgE production by inhibiting the IL-4 produced by Th2 cells (7). In contrast, when stimulated by antigens, mast cells, eosinophils, and non-professional antigen-presenting cells can produce large quantities of IL-10 and IL-4 that block IL-12 and IFN-γ secretion, thereby inhibiting the shift of Th0 cells toward a Th1 phenotype (8). Thus, IFN-γ and its associated cytokines are important in attenuating AR pathogenesis.

Deoxyribonucleic acid (DNA) methylation is an important epigenetic modification. The mammalian DNA methyltransferase (DNMT) family consists of three methyltransferases, DNMT1, DNMT3A, and DNMT3B, which catalyze DNA methylation. Of these enzymes, DNMT1 primarily maintains DNA methylation. It methylates the hemi-methylated DNA formed after replication *in vivo* or *in vitro* such that the methylation level of genomic DNA is maintained. Abnormal activation and overexpression of DNMTs have been found to be closely related to the occurrence and development of various human malignant tumors. The loss of DNMT activity can inhibit or reverse methylation-induced tumor suppressor gene silencing.

Xingbi gel is a medication applied locally to the nasal cavity that was formulated by Huang Shoulin, the director of the People's Hospital affiliated with the Fujian University of Traditional Chinese Medicine, after many years of clinical experience. The gel consists of a mixture of four major Chinese traditional herbs. Briefly, 100 g glycerin and 2 g potassium sorbate are dissolved in 800 ml xuchangqing (*Cynanchi paniculatum* radix) extract followed by the addition of 9 g Carbomer 940 and 10 g *Bletilla striata* gum. The solution is incubated overnight, after which 2 g triethanolamine is added to create the gel matrix. Next, 22 g paeonol, the active ingredient of xuchangqing, 45 g cicada slough (periostracum cicadae), 12 g bezoar (calculus bovis), and 2 g of a β-cyclodextrin inclusion complex containing natural borneol (borneolum syntheticum) are combined using the equivalent incremental mixing method and added to the gel matrix. After thorough mixing, distilled water is added while stirring to bring the volume to 1,000 ml (9).

The drug was incorporated into liposomes. The gel used to create the drug forms a thin film on the nasal mucosa, which promotes drug penetration, as due to gravity, dissolved drugs do not remain at the site for long periods of time. Moreover, the formulation enhances pesticide effect and creates a slow and controlled drug release, thereby improving drug bioavailability and reducing the dose. It overcomes the shortcomings of nasal fillers, such as large volume, inconvenience, or poor compliance, as well as that of traditional Chinese medicine, which must be repeatedly decocted and taken frequently. Accordingly, this study explored the effect of Xingbi gel in AR pathogenesis at the level of Th1/Th2 imbalance, the extracellular-signal-regulated kinase (ERK) signaling pathway, and IFN-γ gene methylation.

## Materials and Methods

Male BALB/c mice at ~8 weeks old were obtained from Shanghai Slake Laboratory Animal Co., Ltd. Aluminum hydroxide, ovalbumin (OVA), the ERK inhibitor PD98059, and the DNMT inhibitor 5-azacytidine (5-Aza) were purchased from Sigma (USA). Xingbi gel was acquired from the People's Hospital affiliated with the Fujian University of Traditional Chinese Medicine (Fujian Pharmaceutical Z2011S0006, batch number: 170703). Enzyme-linked immunosorbent assay (ELISA) kits were purchased from Beijing Solebao Technology Co., Ltd. Antibodies against IFN-γ, IL-4 (flow), DNMT1, DNMT3A, DNMT3B, ERK1/2, and phospho-ERK1/2 (p-ERK1/2) were procured from Abcam (USA). Mouse antihuman glyceraldehyde 3-phosphate dehydrogenase antibody was purchased from Zhongshan Jinqiao Company. Finally, a DNA methylation modification kit was obtained from Zymo.

### Mouse Model of AR

The mice were housed under standard conditions of 22–24°C temperature, 50–60% humidity, and 12:12-h dark-light cycles. The method used to induce the mouse AR model has been previously described (9), with some modifications here. The specific steps are as follows:

On days 0, 6, and 13, mice in the AR group were injected intra-abdominally with 15 μg OVA plus 6 mg of aluminum hydroxide (Al(OH)_3_) gel in a total volume of 0.3 ml. The controls were injected with 0.3 ml sterile saline on the same day. From days 20 to 33, the AR mice were locally sensitized to the OVA challenge *via* intranasal perfusion. The concentration of a 14-μl OVA solution was 40 mg/ml/day. The control mice were injected with sterile saline under the same conditions.

For mice in the AR + Xingbi gel group, the Xingbi gel was administered on the second day after the seventh sensitization injection. The mice were lower-head administered, and 50 μl Xingbi gel/nostril was dropped into both nostrils with a microsampler, three times a day for 11 consecutive days. Control mice were injected with sterile saline under the same conditions.

For mice in the AR + PD98059 group, the ERK inhibitor PD98059 was administered at 10 mg/kg/day, while mice in the AR + 5-Aza group were given 2.5 mg/kg of 5-Aza intraperitoneally. The control mice were given corresponding doses of dimethyl sulfoxide.

### Frequencies of Sneezing and Nose Abrasions

Behavioral changes in AR mice were evaluated using the frequencies of sneezing and scratching. These behaviors was observed and recorded within 20 min following the bilateral nasal drip excitation of OVA.

### ELISA

The mice were anesthetized after their symptom behavior scores were recorded. Their blood was collected *via* orbital puncture, centrifuged at 3,000 rpm for 15 min, and the serum was separated. The levels of OVA-specific IgE, IL-4, and IFN-γ were quantified using ELISA in strict accordance with the kit instructions.

### Hematoxylin and Eosin Staining

Following blood collection, the mice were killed *via* cervical dislocation. The nasal mucosal tissues were collected and fixed with 4% paraformaldehyde. The tissue was dehydrated for 72 h, embedded in paraffin, and sectioned. Routine hematoxylin and eosin (HE) staining was performed. Pathological changes in mice nasal mucosal tissues were evaluated under an optical microscope.

### Flow Cytometry

After the mice were euthanized, they were dissected under aseptic conditions, and the spleens were removed and placed in a Petri dish filled with phosphate-buffered saline. Cluster of differentiation (CD)4+ T cells were isolated from mouse spleen lymphocytes and separated using Dynal immunomagnetic beads. The percentages of Th1 and Th2 expression in the CD4+ T cells were detected using flow cytometry.

### Western Blot

The total protein of the nasal mucosa were extracted. After 10% sodium dodecyl sulfate polyacrylamide gel electrophoresis at 120 V, the protein was transferred to a cellulose nitrate membrane. Following membrane transfer, 5% skim milk + tris-buffered saline with Tween (TBST) was used to block the membrane for 2 h. Next, the membrane was incubated with the primary antibody at 4°C overnight. After washing the membrane, the sheep anti-rabbit immunoglobulin G (IgG) antibody labeled with horseradish peroxidase was incubated for 2 h. Finally, the membrane was washed with TBST for 30 min. The washed nitrocellulose membrane was placed onto transparent film, and the chromogenic solution was added, where after a period of time, the chromogenic reaction began. When bands of appropriate brightness appeared, the film was fixed with a fixing solution, cleaned, and then dried. Finally, the film was scanned or photographed for preservation.

### Bisulfite Sequencing

Cell suspensions were collected in Eppendorf tubes. After centrifugation, DNA was extracted, and its purity was quantified. The DNA OD260/OD280 range in each group was between 1.8 and 2.0. The extracted genomic DNA was modified with bisulfite using a Zymo DNA methylation kit. After bisulfite modification, non-methylated cytosine in 5′–C—phosphate—G-−3′ (CpG) islands as well as all cytosines outside the CpG islands were converted to uracil, which was then converted to thymine when the DNA was amplified *via* polymerase chain reaction. However, methylated cytosine in the CpG islands remained as cytosine. Plasmids extracted from the amplified DNA were sequenced, with the results analyzed using QUMA (10), an online bioinformatics tool.

Gene promoter methylation levels are expressed as methylation rates: average promoter methylation rate = (the number of CpGs methylated by this promoter / the total number of CpGs in the promoter) × 100%; specific CpG site methylation rate = (the number of CpGs methylated at a single CpG site/the CpG site repeat sequencing sample size) × 100%.

### Statistical Analysis

SPSS v17.0 software was used to analyze the data. Analysis of variance was used to compare multiple groups, with Dunnett's T3 test used for variance heterogeneity. Independent *t*-tests were used to compare promoter average methylation rates between two samples, a correction test was used for variance heterogeneity. *P* < 0.05 was considered statistically significant.

## Results

### Behavioral Changes and IgE Reduction in AR Mice

**Figure 1:**
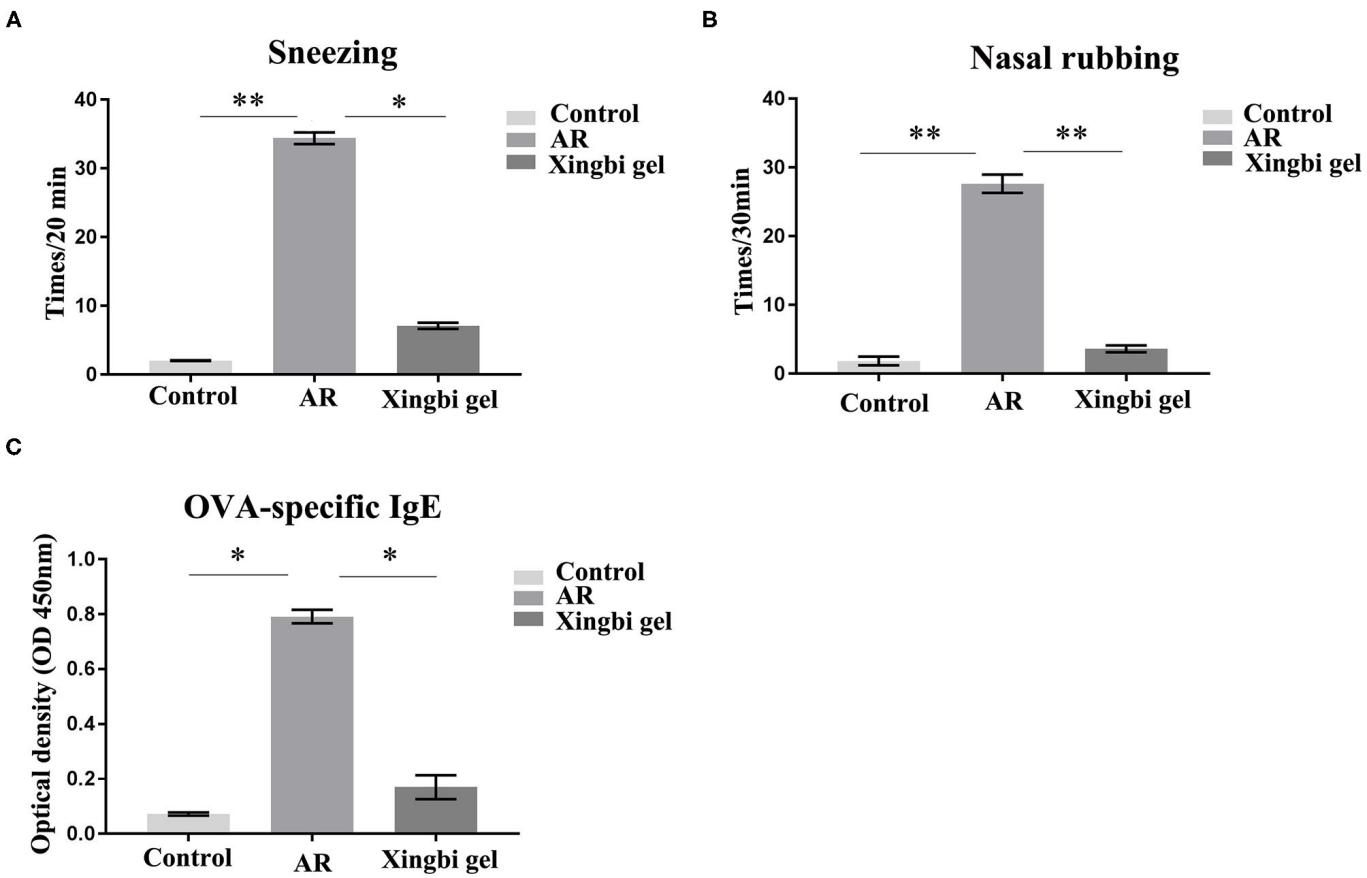
The frequency of sneezing and scratching and IgE content in AR mice increased. **(A)** Compared with the control group, the number of sneezes in AR group increased significantly, and the number of sneezes decreased after treatment with Xingbi gel; **(B)** Compared with the control group, the number of nasal scratching in AR group increased significantly, and the number of nasal scratching decreased significantly after treatment with Xingbi gel. **(C)** ELISA was used to detect IgE levels in the blood of all mice. Compared with the control group, IgE levels in the AR group were significantly increased, while IgE levels in the Xingbi gel group were decreased. ^**^P < 0.01; ^*^P < 0.05.

The frequencies of sneezing and scratching were significantly increased in AR mice within 20 min of OVA excitation compared with the control group. After Xingbi gel administration, the number of sneezes (27.4 ± 1.98 vs. 3.68 ± 0.25 times/20 min) and scratches (24.75 ± 1.77 vs. 1.75 ± 0.35 times/20 min) were significantly decreased (Figures 1A,B).

IgE technology has developed rapidly in recent years. The ELISA method can quantify IgE levels in a more sensitive and rapid manner than the skin and nasal tests, and because it avoids subjective scales, it is also more reliable and accurate. Thus, ELISA was used to quantify OVA-specific IgE in blood obtained from orbital punctures. The results indicate that Xingbi gel can reduce the levels of OVA-specific IgE (0.56 ± 0.13 vs. 0.06 ± 0.02; Figure 1C).

### Effects of Xingbi Gel on the AR Mouse Model

**Figure 2:**
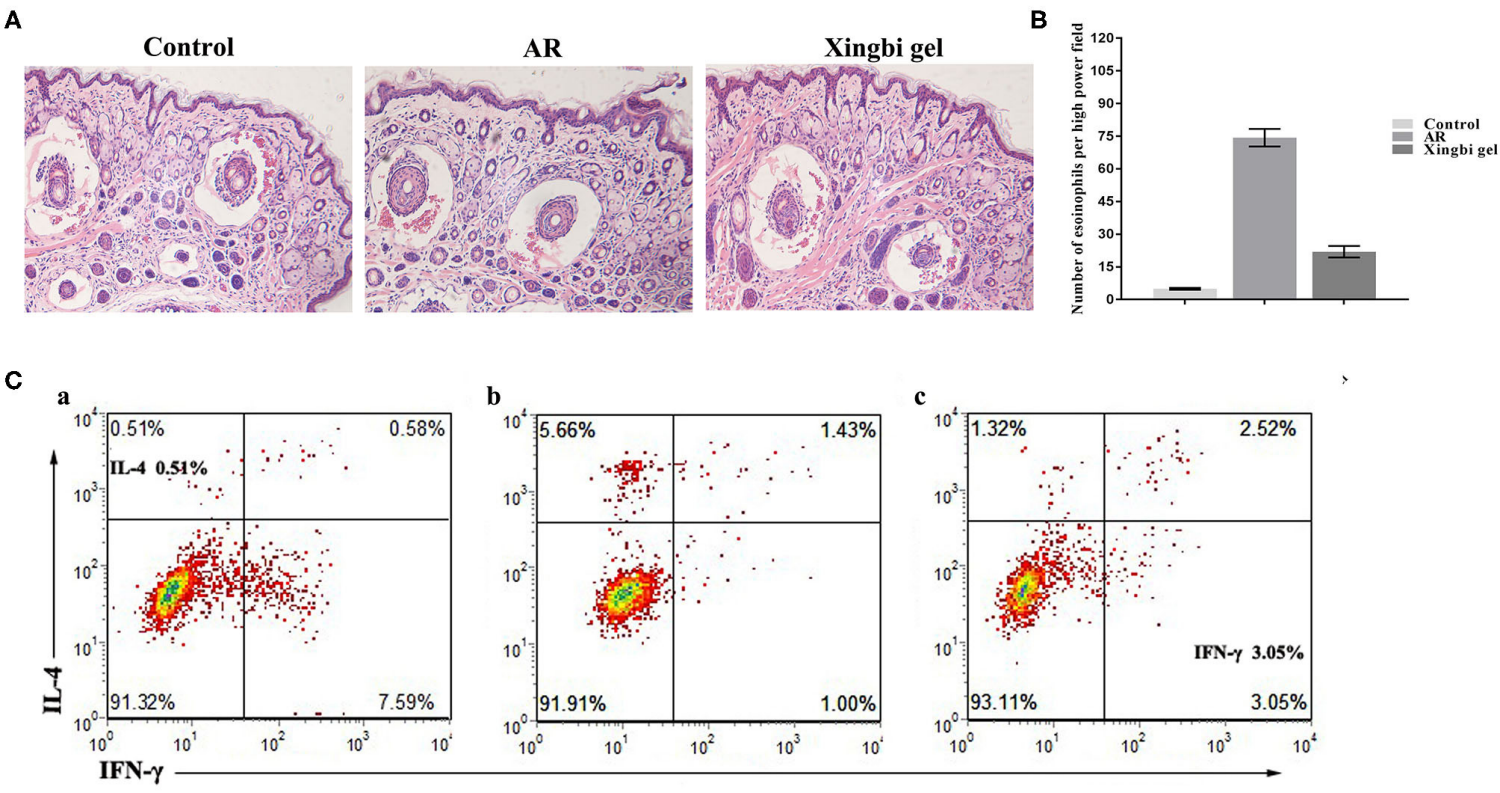
Effects of Xingbi gel on AR mouse model. **(A)** Effect of Xingnin gel on pathological changes. **(B)** Eosinophils histogram. **(C)** Changes in the percentage of Th1 and Th2 expression in CD4+T cells detected by flow cytometry. **(a)** The percentage of IL-4 and IFN-γ in the normal group. **(b)** The percentage of IL-4, IFN-γ and TH1/TH2 in the AR model group was increased, decreased, and the percentage of TH1/TH2 was unbalanced compared with the normal group. **(c)** Compared with the AR model group, the percentage of IL-4 and IFN-γ were decreased and increased after Xingbi gel treatment.

HE staining showed that the nasal mucosa structure of control mice was normal with no observed eosinophil infiltration (Figures 2A,B). However, the nasal mucosa tissues of AR mice were damaged, with the nasal mucosa vessels dilated and edematous and accompanied by a large number of eosinophils. Compared with AR mice, the inflammatory response of the nasal mucosa in mice from the Xingbi gel group was significantly reduced; their mucosal blood vessels were slightly dilated with little edema. Accordingly, the Xingbi gel can ameliorate damaged tissue in AR mice.

Following IFN-γ and IL-4 staining, the percentages of Th1 and Th2 expression in CD4+ T cells isolated from the spleen were detected using flow cytometry. Compared with the control group, the AR mice had a higher IL-4 ratio, a lower IFN-γ ratio, and a Th1/Th2 imbalance. After treatment with Xingbi gel, however, the percentage of IFN-γ increased, while the percentage of IL-4 decreased (Figure 2C).

### Expression Levels of DNMT1, DNMT3a, DNMT3b, ERK1/2, and p-ERK1/2 in CD4+ T Cells From Each Treatment Group as Quantified by Western Blotting

**Figure 3:**
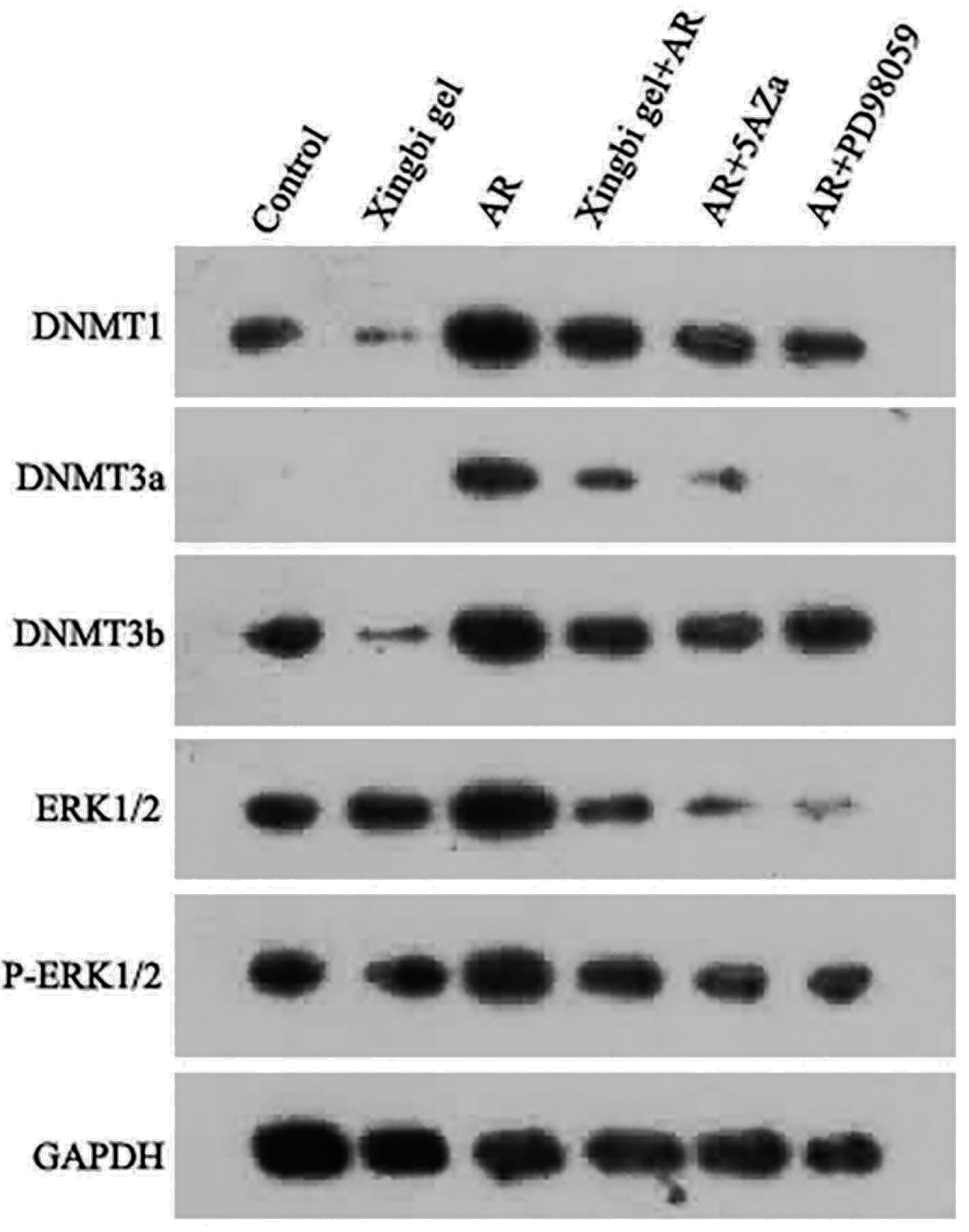
Expression differences of DNMT1, DNMT3a, DNMT3b, ERK1/2 and P-ERK1/2 in AR mice.

In AR mice treated with the Xingbi gel, the expression levels of DNMT1, 3A, and 3B were reduced in CD4+ T cells with no changes in ERK1/2 and p-ERK1/2 expression (Figure 3). 5-Aza reduced the expression of DNMT1, 3A, and 3B in the AR group but also had no effect on ERK1/2 and P-ERK1/2 expression (Figure 3). However, PD98059 reduced ERK1/2 phosphorylation in CD4+ T cells as well as the expression levels of DNMTs in the AR-treated group (Figure 3).

### The Methylation Level of the IFN-γ Gene Promoter in Each Treatment Group as Detected by Bisulfite Sequencing

**Figure 4:**
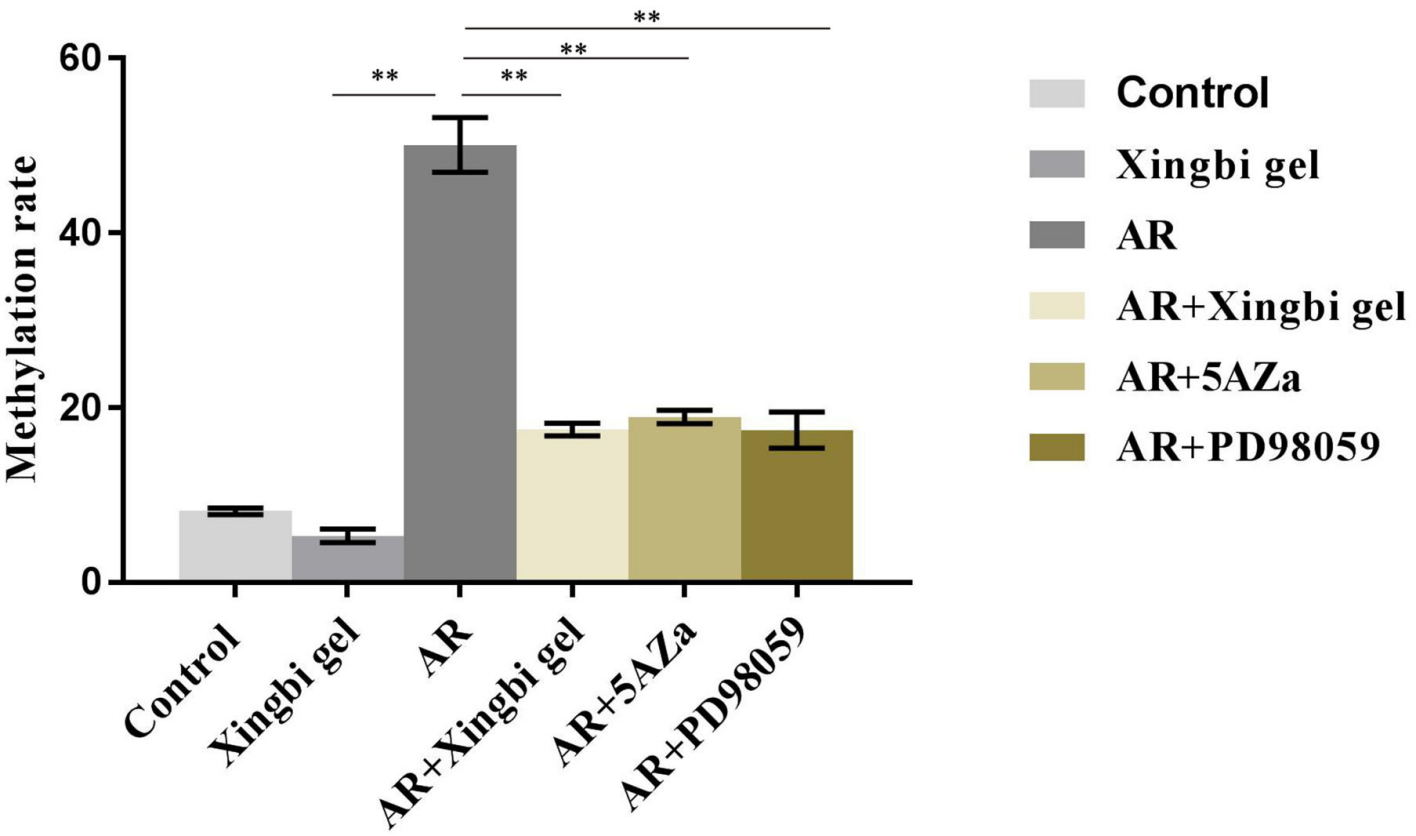
Xingbi gel, PD98059 and 5Aza reduces the IFN-γ gene promoter methylation level.

The promoter methylation rate of the IFN-γ gene was significantly lower in the AR group than in the control group. After treatment with Xingbi gel, PD98059, or 5-Aza, the promoter methylation rate of the IFN-γ gene was significantly higher in AR mice compared with the controls (Figure 4).

## Discussion

AR is a disease involving multiple factors and mechanisms. Regardless of its severity, it can seriously affect the quality of life of its sufferers; for example, patients are often susceptible to fatigue and daytime sleepiness, cognitive impairments, and sleep disorders. These issues can affect a patient's mental state, sleep, personality, and emotions, leading to reductions in job productivity or failing grades. Perennial AR can also induce sinusitis, pharyngitis, otitis media, or bronchitis. With development and progress in scientific research, advances in the basic research and clinical application of AR have been achieved. However, it is challenging to study and treat AR pathogenesis. At present, some progress has been achieved in understanding AR pathogenesis, such as Th1 and Th2 cell differentiation, the role of numerous cytokines, IgE and its receptors, and the hygiene hypothesis. T cells do not produce cytokines in their quiescent state; they can only produce them after activation. When allergens re-enter the nasal mucosa, they are processed to activate T cells, which eventually leads to the secretion of large quantities of respective Th1 and Th2 cell cytokines. The key cytokines are IL-4, which specifically induces IgG and IgE production by B cells, and IFN-γ, which antagonizes IL-4 expression and bioactivity. When IgE binds to basophils and mast cells, a sensitized state is formed, and a series of primary and secondary mediators are released by the IgE receptor. Consequently, glandular secretions are increased, resulting in clinical symptoms that lead to the onset of AR. An imbalance in Th1 and Th2 cell expression has been suggested as an important immunological basis for AR pathogenesis, particularly in the dominant expression of Th2 cells.

Ceuppens believed that CD4+ T cells and their related cytokine products are instrumental in the occurrence and development of AR (11). *In vivo*, T cells are the most important cells involved in immune regulation. Depending on the surface markers and functional characteristics, they are divided into CD4+ and CD8+ T cells. The Th1 and Th2 subsets of CD4+ T cells are divided according to their cytokine secretions and functions. Under the stimulation of specific antigens, Th0 cells can develop into Th1 or Th2 cells (12). IFN-γ, IL-2, IL-12, and IL-7, which are primarily secreted by Th1 cells, are mostly involved in the immune response related to local inflammation. They promote the formation of macrophage-mediated cellular immunity and delay hypersensitivity inflammation. In contrast, Th2 cells mainly secrete IL-4, IL-3, IL-6, and IL-10 to stimulate the proliferation of activated B cells, to produce antibodies, and to participate in humoral immune-related or allergic diseases (13). The main secretion of Th1 cells, IFN-γ, not only transforms the immune response to the Th1 phenotype but also inhibits Th2 cell proliferation and enables static CD4+ T cells to differentiate into Th1 cells (14). Under normal conditions, Th1 and Th2 cells coordinate and antagonize each other through different immune regulatory channels and maintain a dynamic state of equilibrium, which is reflected in the levels of IFN-γ and IL-4 (15).

DNA methylation is an important epigenetic mechanism regulating transcription and maintains normal physiological activity in cells. The lower the CpG island methylation level, the more a gene will be expressed; conversely, the higher the methylation level, the more a gene is silenced (16). In promoter regions, CpG island methylation levels highly correlate with gene transcription inhibition (17). Changes in methylation patterns may lead to the occurrence and development of disease. DNMT catalyzes and maintains DNA methylation. DNMTs, which include DNMT1, DNMT3A, and DNMT3B, can methylate CpG islands at core sequences of structural gene promoters and transcription initiation points, suppress transcription, and shut down the expression of related genes. Notably, DNMT1 is closely related to the state of DNA methylation (18). In this study, in AR mice, the promoter region of IFN-γ, a methylation-sensitive immune-related gene, was found to be hypermethylated; its expression was low. The expression levels of DNMT1, DNMT3A, and DNMT3B in the AR group were reduced by the Xingbi gel and 5-Aza compared with the control group. Thus, the Xingbi gel could reverse IFN-γ hypermethylation. This finding implies that the abnormal function of T cells in AR mice may be related to DNA hypermethylation.

The methylation of genomic DNA can result from the abnormal expression of various signaling pathways such as the ERK pathway. The ERK signal transduction pathway is an important member of the mitogen-activated proprotein kinase family. It regulates numerous cellular processes including gene expression, cell proliferation, cell differentiation, and apoptosis and plays an important role in the pathogenesis of diseases such as cancer, those related to the immune system, and inflammation. The ERK signaling pathway was recently reported to strongly regulate DNMT expression (19, 20). More specifically, in autoimmune disease, ERK signaling was found to be perturbed, resulting in decreased DNMTl expression. Consequently, DNA methylation was reduced, leading to the enhanced expression of methylation-sensitive immune-related genes, which then induced reactive T cells, ultimately leading to the development of autoimmune disease (21, 22).

In this study, the mechanism of T cell dysfunction was examined from an immunological and epigenetic perspective. In transgenic mouse models, DNMT1 expression can be decreased, leading to the overexpression of methylation-sensitive genes. The production of antidouble-stranded DNA antibodies can be induced by disrupting ERK signaling in T cells (22, 23). Indeed, dysfunction in the ERK signaling pathway in T cells have been identified in autoimmune diseases such as systemic lupus erythematosus, and this perturbation is believed to result from damage caused by the activation of T cell protein kinase C (24). These data suggest that genomic DNA is hypomethylated in T cell-associated autoimmune diseases, and the hypomethylation may be related to the deletion or inactivation of the ERK signaling pathway (25). In this study, ERK1/2 and p-ERK1/2 expressions were significantly increased in the CD4+ T cells of AR mice, suggesting that the ERK signaling pathway in these cells is involved in the occurrence and development of AR. The results also imply that DNMT expression is increased. To further evaluate the correlation between the ERK signaling pathway and DNMT expression, AR mice were treated with PD98059, a specific inhibitor of the ERK1/2 signaling pathway. The expression of p-ERK1/2 and DNMTs in these mice was significantly downregulated, a finding previously confirmed in many studies (26, 27). This result suggests that defects in ERK signaling are positively correlated with the downregulation of DNMT1 expression. Combined with the other results from this study, DNMT1 transcription levels in AR mice were positively correlated with overall DNA methylation levels. The promoter region of the methylation-sensitive immune-related gene IFN-γ was hypermethylated and negatively correlated with its transcriptional expression. Therefore, ERK signaling pathway dysfunction likely drives the hypermethylation of CD4+ T cells in the peripheral blood of AR mice.

Baicalin has been reported to have a ameliorating effect on OVA-induced AR guinea pigs by improving rhinitis symptoms and inhibiting the release of inflammatory mediators. It suppresses the production of inflammatory mediators by blocking the Janus kinase 2/signal transducer and activator of transcription 5 (JAK2/STAT5) and nuclear factor kappa-light-chain enhancer of activated B cell (NF-κB) signaling pathways in activated mast cells. Although additional research is required, baicalin may prove to be effective in treating AR (28). The main objectives of AR treatment are to relieve clinical symptoms as much as possible, to prolong the clinical asymptomatic period, to protect nasal mucosa tissue, and to prevent the occurrence of asthma. Baicalin is administered orally, while the Xingbi gel is a concentrated solution that acts directly on the nasal mucosa thereby minimizing any systemic effects. In this study, Xingbi gel nose drops were demonstrated in an animal model that it can improve clinical symptoms by inhibiting IgE expression. Another study (9) found that Xingbi gel can ease typical AR symptoms; the stable expression of mast cells plays a role in inhibiting AR inflammatory responses in mice, improves local/systemic inflammation, relieves sensitization state, prevents the release of inflammatory mediators, and reduces serum IL-4 content. The HE-stained pathological sections also revealed that Xingbi gel decreased the release of inflammatory factors, edema, vascular permeability, and other symptoms caused by AR. Clinical and animal studies have also demonstrated that Xingbi gel nose drops can reduce serum IgE content. Additionally, Xingbi gel can reduce DNA methylation levels, which are negatively correlated with DNMT expression, thereby changing the high methylation state of the promoter regions of the methylation-sensitive immune-related gene IFN-γ. Altogether, Xingbi gel may lower serum IgE levels, adjust the Th1/Th2 balance, regulate the ERK signaling pathway, reduce DNMT expression, and change IFN-γ methylation levels, ultimately ameliorating AR.

## Data Availability Statement

The raw data supporting the conclusions of this article will be made available by the authors, without undue reservation.

## Ethics Statement

The animal study was reviewed and approved by the Ethical Board of Fujian University of Traditional Chinese Medicine.

## Author Contributions

SA performed the majority of the experiments, analyzed the data, and wrote and edited the manuscript. YL analyzed the data and assisted with manuscript revisions. JZ collected and processed the clinical samples. Finally, XZ directed the study, analyzed and approved all the data, and wrote and edited the manuscript. All authors reviewed the manuscript.

## Conflict of Interest

The authors declare that the research was conducted in the absence of any commercial or financial relationships that could be construed as a potential conflict of interest.
